# Experiences of academic and professional burn-out in medical students and residents during first COVID-19 lockdown in Belgium: a mixed-method survey

**DOI:** 10.1186/s12909-022-03694-z

**Published:** 2022-08-20

**Authors:** Issrae El Mouedden, Catherine Hellemans, Sibyl Anthierens, Nele Roos Michels, Ann DeSmet

**Affiliations:** 1 Faculty of Psychological and Educational Sciences, Université Libre de Bruxelles, Avenue Franklin Roosevelt 50, 1050 Brussels, Belgium; 2 Department of Family Medicine and Population Health, Faculty of Medicine and Health Sciences, University of Antwerp, Doornstraat 331, 2610 Antwerp, Belgium; 3 Department of Communication Studies, University of Antwerp, Sint-Jacobstraat 2, 2000 Antwerp, Belgium

**Keywords:** Burnout, COVID-19, Medicine, Student, Academic, Resident

## Abstract

COVID-19 has presented a substantial burden on students and healthcare staff. This mixed-method, descriptive and correlational study aimed to: 1) describe academic; and 2) professional burnout levels; 3) their associations with working in COVID-19-related care; and 4) with perceived COVID-19 impact on studies and internships among medical students and residents. We hypothesized burnout levels to be high; those involved in COVID-19 care to experience higher impact of COVID-19 on studies and work, and to experience higher levels of academic and professional burnout than those not involved in COVID-19 care; academic and professional burnout to be higher when perceived burden due to COVID-19 was higher. During first lockdown in Belgium, a mixed-method cross-sectional survey assessed academic burnout (MBI-SS) and professional burnout in relation to internships and residency (MBI-HSS). Correlations and t-tests tested associations of burnout with involvement in COVID-19-related care and perceived impact of COVID-19 on studies and work (SPSS). Participants provided open-ended comments which were thematically analysed (NVivo). In total, 194 medical students and residents participated (79.5% female, M age = 24.9 ± 2.5). Emotional exhaustion and depersonalisation were high in professional burnout, but moderate in academic burnout. Those involved in COVID-19 related care perceived a higher impact of COVID-19 on their studies and internship/residency and have higher professional burnout, but do not show a higher academic burnout. Those who have a higher perceived impact of COVID-19 on their studies scored higher on academic burnout. Participants mentioned an increased workload (e.g., having to be constantly available and constantly adapt), distress (e.g., uncertainty, fatigue, fear for impact on significant others), fewer learning opportunities (e.g., cancelled internships, changing learning methods), lack of relatedness with patients and supervisors (e.g., lack of respect and understanding from supervisors, distance created by phone consultations with patients) and cynicism towards remote care or non-medical tasks (e.g., considering what they do is not useful or not what they trained for). Students and residents showed indications of professional and academic burnout in relation to the COVID-19 situation. Interventions are needed that can meet the needs of achieving learning outcomes, managing extreme situations and relatedness.

**Supplementary Information:**

The online version contains supplementary material available at 10.1186/s12909-022-03694-z.

## Introduction

The International Classification of Diseases considers burnout as a syndrome related to life-management difficulties and defines it as a state of vital exhaustion [1], that appears in a situation of chronic emotional stress [2]. Burnout can occur in different contexts, including professional (professional burnout) and learning contexts (academic burnout). Professional burnout is characterized by 1) emotional exhaustion, i.e., an emotional fatigue that is not overcome by rest, 2) depersonalisation, i.e., a defensive mechanism that makes it difficult to stay connected to others and can result in detachment and dehumanisation, and 3) a lack of personal accomplishment, i.e., a feeling of inefficacy [3]. Academic burnout refers to the consequences of course load and stress that students experience in the learning process which leads to emotional exhaustion, depersonalisation or cynicism and reduced personal accomplishment [4]. Despite their similarity in dimensions, separate scales exist to measure professional [5] and academic burnout [6] that consider their context specificity and distinguish between experiences students may have on the job (e.g., internships) and during their studies (in relation to their academic activities).

### Theoretical framework

The Job Demands-Resources theory (JD-R) theory states that worker wellbeing results from a balance between demands and resources [7]. When work or study demands exceed the available resources basic psychological needs of competence, relatedness and autonomy are likely to be unmet [8]. Job demands are typically workload, tasks interruptions, organizational changes, and emotional demands. When job demands are high, additional efforts are usually made to try to meet demands, potentially leading to fatigue and exhaustion. Such high demands eventually lead to health erosion such as a burnout syndrome, if a person cannot recuperate from these demands by taking a break or taking their minds off the job by focusing on something else. Job resources, on the other hand, can help reduce these demands or to better manage them. These include personal growth, providing social support, positive feedback and effectively using one’s skills, which can increase basic need satisfaction, job engagement and can reduce the risk of burnout. When resources are low, people tend to motivationally disengage from the job as a mechanism of self-protection [7]. If job resources are low while job demands are high, the risk of burnout increases [9]. Job demands that exceed resources are especially related to the burnout dimension of emotional exhaustion [9].

### Professional burnout in medical professions and training

Especially medical professions appear at risk for burnout, with estimated prevalence figures of 49% among physicians in meta-analytic findings [10]. Prevalence figures, however, largely differ due to variations in definitions, measurement methods and country-specific factors [11]. Students can enter medical training in Belgium after passing a national entrance exam. The training is organised so that students have a basic medical training consisting of a bachelor’s and master’s degree education (each 3 years) [12]. The bachelor’s years consist mainly of organ-focused theoretical courses that address physiological processes. During the master’s years, physiopathology is addressed, again organ-based, in combination with more clinical exposure. Students complete several internships and apply for an advanced master’s education after their master’s degree. During the advanced master’s education, the training and learning is mainly workplace-oriented (‘residency’), in combination with academic activities (e.g., courses, peer-seminars, master thesis). This advanced master’s education (e.g., general practice, specialty medicine, public health, sports medicine) is mandatory to practice as a professional physician. Its duration can range between 3 to 6 years, depending on the chosen specialisation. Medical students who are performing internships and medical residents may thus be exposed to a risk of experiencing both academic burnout related to their studies, as well as professional burnout related to their work with patients during their internships or supervised professional practice. Distinguishing between burnout related to student tasks and occupational life can help inform more targeted interventions in the learning and work environment.

Prevalence rates of academic burnout in medical students ranged from 26 to 45% in cross-sectional surveys [13–16] (using the Maslach Burnout Inventory-Student Survey (MBI-SS)). Prevalence of professional burnout among medical students ranged between 7–75% in systematic review and meta-analytical data [17–19]. Prevalence rates widely vary according to definition, measurement, and cross-cultural differences. Burnout among medical students has a substantial impact on their physical and mental health, well-being, quality of care and future career [20–24] and is therefore important to address early on to avoid detrimental effects on their mental health and later professional career.

### Burden of COVID-19 in medical professions and students

It is generally reported that the COVID-19 pandemic has created a tremendous burden for healthcare staff [25–27]. COVID-19 is a disease that results from an infection with the Sars-COV-2 virus, which is transmitted through close contact and primarily affects the respiratory system [28]. Given the surge in risk factors of an increased workload and reduced level of control, one may expect to see an increase in burnout among medical staff during the COVID-19 pandemic. So far, inconsistent results have been reporting with some studies showing higher burnout scores among healthcare staff and medical resident who were in direct contact with COVID-19 infected people [29, 30], some reporting lower [31–33], and some reporting no significant differences [34]. COVID-19 has not only impacted the work environment but also the learning environment. Particularly among master students [35] and final year students [36], academic burnout rates were high during the COVID-19 period that was characterized by online teaching. Fearing fewer learning opportunities at a moment crucial for their future career is mentioned as a potential reason. Another study, however, indicated lower rates of burnout when teaching switched from live methods to online teaching [37].

### Aims of this study

Given the increased risk of academic and professional burnout among medical students and residents, and the importance of this issue for their mental health and future career, this study aimed to assess the experiences of burnout among Belgian medical students and residents during the first lockdown in Belgium (22 April–4 May 2020). Belgium on average had between 150 and 200 COVID-19-related hospital admissions per day during that period [38].

## Methods

### Research questions

The following objectives and research questions were addressed:

- 1) What are the levels of academic and professional burnout (emotional exhaustion, depersonalisation and reduced personal accomplishment) among medical students and residents during this period? (Research question RQ1);
- 2) What is the difference in perceived impact of COVID-19 on studies and work, and academic and professional burnout levels between those involved/not involved in COVID-19 patient care? (RQ2);
- 3) What is the correlation between academic and professional burnout levels and perceived burden due to COVID-19? (RQ3);
- 4) What are the personal experiences of students and residents in how the pandemic affects their studies and supervised professional practice (RQ4)?

**Fig. 1:**
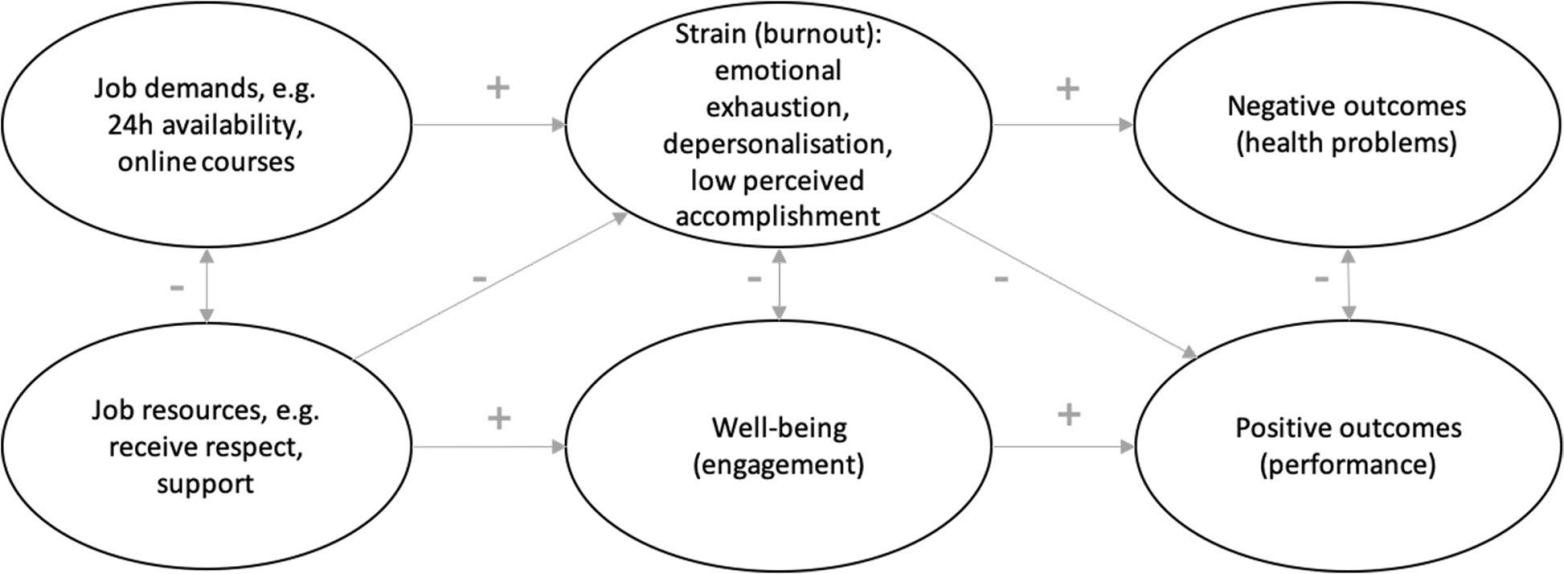
Job demands-resources model [7], applied to academic and professional burnout during COVID-19

Based on the JD-R model (see Fig. 1), we hypothesized that:

- H1) burnout levels would be high;
- H2) that those involved in COVID-19 care, representing a form of increased demands, would experience higher impact of COVID-19 on studies and work.
- H3) and experience higher levels of academic and professional burnout than those not involved in COVID-19 care;
- H4) that academic and professional burnout would be higher when perceived burden due to COVID-19 was higher, as this perceived burden represents an imbalance between demands and resources.

No hypotheses are made for RQ4 which is an exploratory, qualitative analysis of participants’ open-ended answers. We, however, expect to find experiences of increased demands during COVID-19 pandemic, both in academic and work-related life, which according to the JD-R model can lead to higher strain if it is not accompanied by sufficient resources. We will structure the qualitative findings according to these theoretical elements.

### Participants and procedure

The target population for this study were master students in medicine with internship experience and advanced master students in medicine (medical residents). Participants were recruited via convenience sampling, using a link that was published on six social media groups (Facebook) of French-speaking universities in Belgium created by medical students to share academic information. Students use these groups to share available internships, information about courses, to ask specific questions, to give or ask for help on matters regarding their studies. The majority of students are part of these groups. The combined number of members of these groups is *n* = 6918. The announcement asked for their participation in a study on their experiences of academic life. No mention of burnout or stress was made up front. A debriefing was given about the precise research questions and hypotheses after completion of the study. The announcement included a link to the anonymous online survey (programmed in Limesurvey) between 22 April – 4 May 2020, which took 10–15 min to complete. Based on IP address, the survey prohibits multiple responses from the same participant. As no monetary incentive was provided for participation, there was also no encouragement for students to fill out the survey more than once. Participants provided informed consent at the start of the survey. The study was approved by the ethics committee of Université Libre de Bruxelles (035/2020). Inclusion criteria were being able to read and understand French; being a master student in medicine in one of the French-speaking Belgian universities with internship experience or being a medical resident; and having Internet access. Master students with no professional experience (not yet having conducted an internship) were excluded. No financial incentives were provided for study participation. Power analysis using G*Power indicated a sample size of *n* = 77 was required for regression analyses (3 predictors, small to moderate effect size f^2^ = 0.15 as a conservative measure, α = 0.05, 1-β = 0.80); of *n* = 82 for correlations (one-sided testing given the hypotheses, small to moderate effect size of *ρ* = 0.3 as a conservative measure, α = 0.05, 1-β = 0.80); and *n* = 128 for independent sample t-tests (small to moderate effect size of d = 0.50 as conservative measure, α = 0.05, 1-β = 0.80).

### Measurements

#### Profile of participants

The survey assessed sociodemographic characteristics, including gender, age, marital status, and household situation. This part also assessed study characteristics, such as year of study, professional status (internship experience, master student/resident), the ward where they conducted their internship/residency at the time of the survey and the duration of the internship/residency.

#### Academic burnout

Participants were asked to answer the items of the MBI-SS thinking exclusively of their studies. The French version of the Maslach Burnout Inventory Student Survey (MBI-SS) [6] was used in this study to measure burnout levels in an academic context. The MBI-SS has 15 items and three dimensions: emotional exhaustion (EE, 5 items, e.g. ‘I feel emotionally drained from my studies’); academic efficacy (AE, further referred to as PA, personal accomplishment for ease of comparison with professional burnout, 6 items, e.g. ‘In my opinion, I’m a good student’) and the cynicism or depersonalisation dimension (CY, 4 items, e.g. ‘I doubt the significance of my studies’) [6]. All items are evaluated on a 6-point Likert-type scale (1–6). Scores per dimensions are based on the sum of item scores for each dimension. Cut-off points for burnout scores per dimension were drawn from Faye-Dumanget and colleagues [6] (cut-off scores obtained in personal communication with the authors on d.d. 30/03/2020) and used to categorize sum scores into burnout levels per dimension: EE low ≤ 13, moderate 14–22, high ≥ 23; CY low ≤ 10, moderate 11–17, high ≥ 18; PA low ≤ 16, moderate 17–26, high ≥ 27. Higher scores and the category ‘high’ for the dimension of PA reflect a higher perception of accomplishment. Important to note is that in academic burnout, the category ‘high’ for PA is interpreted in a positive manner, reflecting a higher sense of personal accomplishment, whereas for professional burnout, the category ‘high’ on PA reflects high risk for burnout and should be interpreted negatively, as a high reduction in perceived personal accomplishment, in line with the norm scores that are used for each specific scale.

#### Professional burnout

Participants were asked to answer the items of the Human Services Survey for Medical Personnel (MBI-HSS) thinking exclusively in relation to their medical internship. The French version of the Maslach Burnout Inventory Human Services Survey (MBI-HSS) [5] was used. The MBI-HSS has 22 items and three dimensions: emotional exhaustion (EE, 9 items, e.g. ‘I feel emotionally drained from my work’), depersonalisation (DP, 5 items, e.g. ‘I don't really care what happens to some recipients’) and personal accomplishment (PA, 8 items, e.g. ‘I have accomplished many worthwhile things in this job’). Items are evaluated on a 7-point Likert-type scale (from 0 to 6). Scores per dimensions are based on the sum of item scores for each dimension. Cut-off points for burnout scores per dimension for physicians from Maslach et al. [39] were used to categorize sum scores into burnout levels per dimension: EE low ≤ 18, moderate 19–26, high ≥ 27; DP low ≤ 5, moderate 6–9, high ≥ 10; PA (lack of) low ≥ 40, moderate 34–39, high (lack of perceived personal accomplishment) ≤ 33. For the dimension of PA, average scores reflect their perception of accomplishment (a higher score reflects higher accomplishment), whereas the categories reflect lack of PA (the category high reflects lower accomplishment).

#### Involvement in COVID-19 care and perceived impact of COVID-19 on studies and internship/residency

Participants were asked if they had been involved in the treatment of COVID-19 patients during their internship or residency (yes/no). Two questions assessed their perception of the influence of the pandemic on respectively their studies and their internship/residency (e.g., ‘To what extent do you feel that the COVID-19 crisis has impacted your studies’). These items were answered on a 6-point Likert scale (not at all – very much). These questions were followed by an open-ended question to give the opportunity to the participants to explain their experience. All participants were invited to answer the open-ended question that was used for the qualitative analyses.

### Analysis

All quantitative analyses are performed on the scales for each dimension, as the MBI advises against calculating one overall burnout score [40]. Normal distribution of the six continuous dependent variables was checked via histograms, Q-Q plots, kurtosis and skewness information (range of -2; + 2). All variables were normally distributed. Independent samples t-tests were used to examine differences in mean burnout rates between two groups. Summary independent samples t-tests were used to compare levels of burnout with findings reported in literature. Pearson bivariate correlations were used to assess associations between burnout rates and perceived impact of COVID-19. Linear regression analyses were conducted to examine the explained variance of the predictors ‘involved in COVID-19 related care (dummy coded)’, ‘perceived impact of COVID-19 on studies’ and ‘perceived impact of COVID-19 on internships’ on the six burnout dimensions. Significance level was set at 0.05 for all tests. All quantitative analyses were performed in SPSS version 27. Quantitative data are available from the authors upon request. The open-ended answers (RQ4) were analysed via a combination of an inductive and deductive approach in Thematic Analysis [41] using NVivo software (release 1.4.1) to support the coding process. A coding frame was constructed with the three main dimensions of burnout as main themes. Subthemes and additional themes were created based on the meanings that emerged from the respondent’s answers. Themes were reported separately for those in COVID-19-related care and those not involved in COVID-19 care, to better understand potentially different experiences. Quotes of participants were shown mentioning whether participants were master students not in active internship (‘student’), master students in active internships (‘interns’) or advanced master students in residency (‘residents’). Double coding was conducted by an independent second rater for 10% of the material. Cohen’s kappa for double coding = 0.72 (96% absolute agreement) shows substantial agreement between both raters. Findings between coders were discussed to increase coding reliability.

## Results

### Profile of participants

Out of 6918 members of the social media groups, 444 viewed the invitation link (response rate of 6.4%). A total of 194 participants completed the survey (completion rate of 43.7% resulting in a final response rate of 2.8%; 79.5% female, average age = 24.9y ± 2.5). This comprised 36.6% master students who had completed but were currently not in an internship, 38.1% master students in an ongoing internship and 25.3% residents. Of the medical master students, 18.6% were in their fourth year, 26.2% were in their fifth year and 55.2% were in their final, sixth year. The vast majority of students were unmarried (94.5%). Most lived together with their family (40.3%), 26.7% lived together with their partner, 21.5% were sharing a place with others who were not their partner or family, and 11.5% lived alone. The wards where students in internship or residents were currently working were general practice (34.7%), emergency care (20.7%), paediatrics (11.6%), surgery (11.6%), gynaecology (9.1%), psychiatry (5.0%), gerontology (5.0%), anaesthesia (1.7%) and dermatology (0.8%). For most, their current/last internship/residency had lasted less than three months (71.1%), for 1.5% their current/last work experience had lasted between 3–6 months, for 23.7% between 6 and 12 months and for 3.6% more than 12 months.

### Main Results

#### Burnout levels

Descriptive information and correlations for the study variables is shown in Supplementary Material (Suppl. Table 1). Internal consistency of dimensions in our dataset showed *α* = 0.85 for EE, *α* = 0.71 for AE/PA and *α* = 0.84 for CY. All coefficients are above the desirable level of 0.70.

In total, 17.0% of participants had high scores on all three dimensions of professional burnout. The majority showed high levels of professional burnout on the dimension of emotional exhaustion-EE (43.8%), high levels of professional burnout on the depersonalisation-DP dimension (46.9%), and around a third showed high levels of lack of perceived personal accomplishment-PA (36.6%) (Table 1). These results confirm our hypothesis 1, showing high levels of professional burnout. Few participants had high scores on all three dimensions of academic burnout (0.5%), as few participants scored high on cynicism (10.8%). For academic burnout, the majority had moderate levels of emotional exhaustion-EE (67.0%), for cynicism CY/DP (56.2%) and for personal accomplishment-PA (73.2%). The levels of academic burnout thus appear less elevated than those of professional burnout.

**Table 1:** Academic and professional burnout levels in medical students and residents (*n* = 194)

		**Academic burnout** **MBI-SS**	**Professional burnout** **MBI-HSS**
**Emotional exhaustion (EE)**			
*M* ± *SD*		19.15 ± 4.62	25.96 ± 11.35
	Low	9.8%	27.3%
	Moderate	67.0%	28.9%
	High	23.2%	43.8%
**Depersonalisation (DP) / Cynicism (CY)**			
*M* ± *SD*		11.48 ± 4.54	9.87 ± 6.53
	Low	43.8%	28.9%
	Moderate	45.4%	24.2%
	High	10.8%	46.9%
**Personal Accomplishment (PA)**			
*M* ± *SD* (personal accomplishment)		22.12 ± 4.15	34.99 ± 7.39
**Academic PA** **/ Lack of professional PA**	Low	8.2%	33.5%
	Moderate	73.2%	29.9%
	High	18.6%	36.6%

*Legend*: Important to note is that in academic burnout, the category ‘high’ for PA is interpreted in a positive manner, reflecting a higher sense of personal accomplishment, whereas for professional burnout, the category ‘high’ on PA reflects high risk for burnout and should be interpreted negatively, as a high reduction in perceived personal accomplishment, in line with the norm scores that are used for each specific scale.

#### Burnout levels in comparison to literature

A comparison using summary T-Tests with meta-analytic findings of burnout studies among medical students shows significantly higher degrees of EE in our sample than in the meta-analysis (*n* = 12,246 students, *M* = 22.93 ± 10.25, *t*(12,438) = 4.08, *p* < 0.001); significantly higher degrees of DP in our sample than in the meta-analysis (*n* = 12,246 students, *M* = 8.88 ± 5.64, *t*(12,438) = 2.42, *p* = 0.016); but no differences with the levels in PA reported in the meta-analysis (*n* = 12,246 students, *M* = 35.11 ± 8.03, *t*(12,438) = -0.21, *p* = 0.84) [17]. Levels of academic burnout were more moderate. A comparison with a study conducted in 2019, using the MBI-SS scale among medical students in Spain (no comparison in Belgium available) showed significantly lower rates of academic burnout in our sample than in the Spanish sample for EE (*n* = 1073, *M* = 27.50 ± 7.16, *t*(1265) = -15.81, *p* < 0.001), for DP/CY (*n* = 1073, *M* = 14.83 ± 7.09, *t*(1265) = -6.35, *p* < 0.001), but again showed no significant differences for PA (*n* = 1073, *M* = 22.38 ± 6.89, *t*(1265) = -0.51, *p* = 0.61) [42].

#### Perceived impact of COVID-19

There were significant differences (*t*(192) = 2.64, *p* = 0.009) in the perceived impact of COVID-19 on their studies between participants who had provided care to COVID-19 patients (*M* = 4.55 ± 1.38) and those who did not (*M* = 3.96 ± 1.70). Those who were involved in COVID-19 care perceived a significantly higher impact of COVID-19 on their studies than those not involved in COVID-19 care. There were also significant differences (*t*(115) = 4.70, *p* < 0.001) in the perceived impact of COVID-19 on their residency and internships between participants who had provided care to COVID-19 patients (*M* = 4.44 ± 1.49) and those who did not (*M* = 3.24 ± 1.79). Those who were involved in COVID-19 care perceived a significantly higher impact of COVID-19 on their work and internships than those not involved in COVID-19 care. These results confirm hypothesis 2. There were no significant differences in academic burnout between participants who had provided care to COVID-19 patients and who did not (Table 2). Significant differences were found in the level of DP at work, which were higher when involved in COVID-19 care. This provides some support for hypothesis 3 at the level of professional but not academic burnout.

**Table 2:** Academic and professional burnout by those involved/not involved in COVID-19 care

Dimension	Not involved in COVID-19 care (*n* = 67) *M* ± *SD*	Involved in COVID-19 (*n* = 127) *M* ± *SD*	*t* (df), *p*
MBI SS—Emotional exhaustion	19.66 ± 4.39	18.89 ± 4.73	*t*(192) = -1.10, *p* = .27
MBI SS—Cynicism	11.48 ± 4.65	11.48 ± 4.50	*t*(192) = 0.00, *p* = .99
MBI SS—Personal accomplishment	22.00 ± 3.63	22.18 ± 4.41	*t*(192) = 0.29, *p* = .77
MBI HSS—Emotional exhaustion	26.79 ± 11.09	25.53 ± 11.02	*t*(192) = -0.74, *p* = .46
MBI HSS—Depersonalisation	8.54 ± 5.61	10.57 ± 6.88	*t*(159) = 2.22, *p* = .03
MBI HSS—Personal accomplishment	33.67 ± 7.71	35.69 ± 7.14	*t*(192) = 1.82, *p* = .07

#### Correlation between perceived impact of COVID-19 and burnout levels

The perception that COVID-19 had impacted their studies (*M* = 4.35 ± 1.52) was significantly correlated to higher levels of EE related to their studies (*r* = 0.24, *p* < 0.001) and to higher levels of CY related to their studies (*r* = 0.20, *p* = 0.006). Higher scores on perceived impact of the COVID-19 pandemic on their studies was negatively correlated with their perceived academic accomplishment (*r* = -0.15, *p* = 0.04). No significant negative correlation was found between professional DP and perceived impact of COVID-19 on their studies (*r* = -0.14, *p* = 0.06), nor for perceived impact of COVID-19 on their residency/internship (*M* = 4.03 ± 1.70). These findings partially confirm hypothesis 4: significant relations were found between all academic burnout dimensions and perceived impact of COVID-19 on their studies. No relation with perceived COVID-19 impact was found for any of the professional burnout dimensions.

Regression analyses were conducted to assess explained variance of these predictors of burnout. All findings are consistent with the results from the t-tests and correlations. None of the predictors significantly predicted professional emotional exhaustion (F(3,190) = 0.78, *p* = 0.50, adjusted R^2^ = 0.00). The model with three predictors for professional depersonalisation was not significant (F(3,190) = 1.47, *p* = 0.23, adj. R^2^ = 0.01), only the model that contained involvement in COVID-19 related care predicted professional depersonalisation (F(1,192) = 4.35, *p* = 0.04, adj. R^2^ = 0.02). The predictive model for professional personal accomplishment was significant (F(3,190) = 3.19, *p* = 0.03, adj. R^2^ = 0.03), in which only perceived impact of COVID-19 on their studies was a significant predictor (β = -2.43, *p* = 0.02). The model with three predictors was significant in explaining variance in academic emotional exhaustion (F(3, 190) = 5.83, *p* < 0.001, adj. R^2^ = 0.07) in which only perceived impact of COVID-19 on their studies was a significant predictor (β = 0.34, *p* < 0.001). The model with three predictors significantly explained variance in academic cynicism (F(3,190) = 2.78, *p* = 0.04, adj. R^2^ = 0.03) in which only perceived impact of COVID-19 on their studies was significant (β = 2.70, *p* = 0.008). The 3-predictor model did not significantly predict academic personal accomplishment, only a model that contained perceived impact on the studies was significant (F(1,192) = 4.10, *p* = 0.04, adj. R^2^ = 0.02). These results indicate an overall low explained variance of the included variables on burnout dimensions.

#### Qualitative findings on burnout experiences during COVID-19

Table 3 with illustrative quotes shows the themes and subthemes that were created from the analysis of open-ended answers on how the COVID-19 pandemic had impacted their studies and work/internships. Responses to the open-ended question were provided by *n* = 133 participants. A first, most frequently mentioned, overarching dimension is that of emotional exhaustion. Students reported feeling exhausted because the workload had increased due to the COVID-19 crisis. This partly resulted from difficulties in having to combine all tasks involved in their work and education. An increased workload was also experienced as certain tasks had become more demanding and time-intensive and required a constant availability. Moreover, additional or new resources were required by the new format of teaching, which added to the perception of increased workload. At the same time, students felt that insufficient adjustments were made to make this workload manageable. This related to the experience of psychological distress: students mentioned feeling exhausted, uncertain, worried about their family, impacted by the general sense of stress in society, lonely, unmotivated to study and powerless. This psychological distress seemed to be heightened by having to constantly adapt to a changing situation and not being able to take some rest. Some students also nuanced this distress by mentioning that in the light of greater things, they did not feel they had the right to complain.

**Table 3:** (Sub-)Themes in personal experiences of COVID-19 impact on studying and internship

Theme / subtheme	n references COVID care (*n* = 129, 311 references)	n references Non-COVID care (*n* = 67, 113 references)	Quote
Theme 1. Job-study demands			
** Subtheme: Increased workload (general)**	29	3	“*I work at emergency care and the work here is hard. It’s 6/7 days with quite long hours. Leaving home at 6.30AM to arrive home at 7PM*” (COVID care, male, intern)
** Subtheme: Lack of adjustment of requirements to changed context**	8	6	“*The workload has not been adjusted by the faculty and the on-call duties for COVID nevertheless have to be guaranteed*” (COVID care, female, intern)
** Subtheme: More time-intensive, everything takes longer**	4	2	“*Everything is slowed down, we must constantly remind those involved (e.g. lab heads) that we were waiting for their help as agreed … It is very time consuming, while we are all running out of time!*” (non-COVID care, female, intern)
** Subtheme: Having to be constantly available**	4	0	“*In terms of hours, these increased slightly but not unreasonably so. It is however this need to be constantly available that is exhausting*” (COVID care, female, resident)
** Subtheme: Need for constant adaptation in changing context**	5	0	“*The constant adaptation is exhausting* ” (COVID care, female, student)
** Subtheme: Difficulties when studying at home (interference, independent, technical problems)**	8	4	“*While some, who are used to studying in the library, are now obliged to do so from home, sometimes with little brothers and sisters to watch over as the parents have to work*” (COVID care, female, intern)
** Subtheme: Changes in how teaching/exams takes place**	12	5	“*Lack of internship, lack of practice. A decrease in various consultations. Less technical acts. Permanent stress and tangible tension*” (non-COVID care, female, intern)
** Subtheme: More responsibility when on call at internship**	3	1	“*More responsibilities during on-call”* (COVID care, female, intern)
** Subtheme: Change of work content (all focused on COVID-19)**	8	3	“*It disrupted our *“*normal*”* learning. It slowed down all services except emergencies and covid units preventing those in surgery, dermatology, *etc*. for example to learn from their internships*”* (non-COVID care, female, intern)*
** Subtheme: less direct patient contact, teleconsultation and lack of follow up (transformation of care)**	29	6	“*Telephone contacts all day long to “sort out” patients: dehumanization, anxiety, fear of not being up to the task by telephone*” (COVID care, female, resident)
THEME 2: LACK OF RESOURCES			
** Subtheme: Lack of emotional support by university instances**	11	4	“*The faculties did nothing for us except postpone the submission date. Oral defences and exams are maintained with no reduction in material to learn. No discussion possible with them. No empathy. We are treated like beasts*” (non-COVID care, female, student)
** Subtheme: Lack of respect (from supervisors, university, patients)**	7	2	“*This situation was a hard blow for me morally because I had volunteered to help with the conviction that the faculty authorities, made up of caregivers, would understand the situation and would value it by being understanding and indulgent. Almost no understanding was received, however (other than a *“*benevolent indulgence*”* for oral evaluations)*” (non-COVID care, male, intern)
** Subtheme: Promotors who are not available for supervision (sick, exhaustion)**	5	0	“*Our supervisors have relatives with coronavirus and no longer have the desire or the energy to help us … We feel alone and abandoned in this process when we need them most*” (COVID care, female, intern)
** Subtheme: No possibility to unwind (relax, no holidays)**	5	0	“*Very little time to rest”* (COVID care, female, resident)
**Subtheme: Fewer learning opportunities**	6	8	“*Required in the covid units* = *more important and less instructive work than when we are in a classic internship (therefore *“*waste of time*”* from a learning point of view)*” (non-COVID care, female, student)
** Subtheme: Important aspects of education (temporarily) cancelled**	29	25	“*Three internships of one month each were cancelled*” (COVID care, male, student)
** Subtheme: Administrative or nursing work instead of learning as medical intern / redundant work**	11	2	“*The trainees are not used as interns in medicine but as a secretary / nurse / help in caretaking* ” (non-COVID care, female, student)
** Subtheme: Non-functioning or unavailable material during care**	3	0	“*Logistical problems: a single scanner available for all emergencies considered covid including dyspnoea, chest pain and temperature regardless of the context, laboratory slowed down for hygiene reasons, time wasted trying to convince the floors to hospitalize a person because they are all afraid of the covid … Lack of rooms and nursing staff: all treatment is slowed down and patients keep accumulating*” (COVID care, female, resident)
** Subtheme: Considering certain parts of the training or the job irrelevant or absurd**	3	0	“*Emotionally, it’s very difficult to stay at home to work on absurd things like the master thesis, while the medical profession is in distress and we are asked to come and help, every day.*” (COVID care, female, intern)
THEME 3: RESOURCES			
** Subtheme: Positive opportunity to have more time to study due to cancellations of certain tasks**	8	2	“*We were therefore able to study for our exams without stress and do our research for the thesis and for our group work.*” (non-COVID care, female, student)
** Subtheme: No change, all is maintained via digital means**	3	1	“*Identical: personal exercises and exams maintained *via* Teams and online questionnaires* ” (COVID care, female, intern)
** Subtheme: Volunteering (general)**	27	9	“*Less internship activity but compensated by volunteering activity”* (non-COVID care, male, intern)
** Subtheme: Reduced workload in non-COVID-19 care**	11	2	“*Either heavy workload in the units concerned, or lack of work everywhere else*” (COVID care, male, intern)
THEME 4. STRAIN			
** Subtheme: Psychological distress (general)**	13	3	“*The psychological stress associated with the epidemic greatly increases the workload because it is about working against one’s own best interest when caring for these patients”* (COVID care, female, resident)
** Subtheme: Stress related to having to combine everything**	12	4	“*Students are asked to help in COVID units and this takes time, knowing that we also need to work on our master thesis and final exams that have not been cancelled”(non-COVID care, female, student)*
** Subtheme: General stress in relation to COVID-19 (impact on family, environment at work)**	11	5	“*The workload is not too much of the problem. Rather, it's the emotional load, the environment of constant anxiety, not seeing friends and losing my mind, that tires me at the moment*”* (non-COVID care, female, resident)*
** Subtheme: Uncertainty**	6	6	“*We have to deal more with the unexpected than when our days are already well organized in advance*” (COVID care, female, resident)
** Subtheme: Fatigue, exhausting**	5	1	“*It affects my morale and therefore my impression of having to give all my energy to perform simple tasks*” (COVID care, female, student)
** Subtheme: Loneliness, missing social contact**	2	4	“*To not see your mates during a long time makes you lose your energy*” (non-COVID care, female, student)
** Subtheme: Feeling of being powerless, lack of control, no perspective of improvement**	4	0	“Psychological *burden* + + + *because high mortality and powerlessness*” (COVID care, male, student)
** Subtheme: Feeling of not being allowed/supposed to complain**	2	1	“*Not ideal, but nothing to complain about either *” (COVID care, male, student)
** Subtheme: Lack of motivation to study, cannot concentrate**	6	3	“*I was much less motivated to study, and the various adjustments it caused were very stressful*” (non-COVID care, female, student)
** Subtheme: General stress facing patients**	10	1	“*It is also necessary to manage the stress of patients, reassuring them is energy consuming*” (COVID care, female, resident)

A second overarching dimension is that of perceived personal accomplishment. With changes in learning modalities, cancellations of certain parts of teaching or internships, and a narrowed focus of learning content on COVID-19, students felt they had fewer learning opportunities. They worried about missing crucial elements in their training. Several mentioned having taken up volunteering activities to help manage this crisis, and some reported taking part in volunteering to compensate for cancelled learning opportunities.

A third overarching dimension reflected interpersonal issues of depersonalization and lack of relatedness. Several students noted a change in how care was organised, with more phone consultations and fewer face-to-face contact with patients. Some reported that in their opinion this was not good practice. They feared missing the skills on how to effectively manage patients’ needs remotely. Contact with patients was also characterized by stress about managing patients’ anxieties. At an interpersonal level, students moreover stated feeling let down by the university: they felt the university instances did not show enough understanding or respect or were not sufficiently available for support. Students were investing a lot of time and effort into volunteering and helping to manage the crisis, some were studying in difficult situations or experienced a lot of distress, for which the university showed insufficient recognition.

A final, fourth, overarching dimension is that of cynicism: students were doubting the sense of what they were doing during their studies or internship. They felt that their work was reduced to performing administrative or nursing tasks. They questioned the quality of care or the relevance of focusing on their studies while a more urgent need was experienced for help in the hospitals. Some participants, however, did not experience an increased workload, exhaustion, depersonalisation or change in personal accomplishment. They had the impression that the cancelled parts of their training had freed up time for other tasks, or mentioned that all training was maintained, albeit in a different format. Certain differences in emphasis were found between those in COVID-19 related care and those not working in COVID-19 care.

## Discussion

### Burnout levels

The aggregated levels of burnout among master and advanced master students in medicine were moderate: 17% of participants scored high on all dimensions of professional burnout and < 1% scored high on all dimensions of academic burnout, due to a low number of participants with high scores on the dimension of cynicism. These levels of burnout seems low compared to 44-51% of overall professional burnout rates in meta-analyses among medical students and residents [18, 19] and to ranges of 26-45% of aggregated academic burnout rates among medical students and residents [13–16]. However, this may be due to different calculation methods. Scores are more in line when comparing to similar calculation methods. A summary table of these comparisons is available in Supplementary material. Moreover, it is important to mention that norm scores cut-off points may differ by professional population and between cultures. Rather than interpreting aggregated burnout rates, it is therefore recommended to examine varying degrees of burnout per dimension [40]. Ideally, norm scores are used that are specific to a certain professional and national context [43]. As no MBI norm scores were available for physicians or medical students in Belgium, US norms for health service workers were used for the MBI-HSS [39] and French norms for university non-medical students for the MBI-SS [6].

### Burnout levels in comparison to literature

Levels of the various burnout dimensions in our sample showed higher levels on professional emotional exhaustion and depersonalisation/cynicism than in a meta-analysis among medical students, but no difference on personal accomplishment [17]. Conversely, levels of academic emotional exhaustion and depersonalisation/cynicism were lower in our sample than in a Spanish study among medical students, but again showed no differences in personal accomplishment [42]. Considering limitations of cultural differences and that these comparison studies were conducted outside of the context of COVID-19, it may be prudent to conclude that the professional burnout dimensions of emotional exhaustion and depersonalisation/cynicism are relatively high in our study, and that academic burnout dimensions of emotional exhaustion and depersonalisation/cynicism are relatively low. At the time of first COVID-19 lockdown, some teaching activities were halted, which may have led to a lower level of academic emotional exhaustion. Internships and residency as professional activities may, nevertheless, have become more demanding and have increased professional emotional exhaustion. However, many participants in our study mentioned the halting of teaching activities as a source of stress rather than a relief. Cynicism and depersonalisation in relation to their studies seem low. The COVID-19 pandemic may have confirmed and strengthened students’ conviction that their medical studies are important. This would contradict findings from a Canadian survey among medical students that showed one in five were reconsidering their career choice [44]. Depersonalisation, however, seemed high for their internships and residency. In relation to these, participants mentioned not being convinced of the quality of telephone consultations, experiencing a lack of contact with patients, and seeing their work reduced to non-medical tasks. This inconsistency between role expectations and their tasks asked to perform, may also reflect a questioning of their ‘professional’ identity.

### Perceived impact of COVID-19 and its relation to burnout

A greater impact of COVID-19 on their studies and their internships/residency was perceived by students who were involved in COVID-19-related care, and this perceived impact correlated significantly with higher burnout rates on all dimensions for their studies. This was not the case for their internships or residency. Those who were involved in COVID-19-related care experienced a higher level of depersonalisation or cynicism in their internships or residency. No differences were found in academic burnout by their involvement in COVID-19-related care. These results confirm other study findings that professional burnout rates are higher among those who work as COVID-19 frontline workers [29, 30].

Our study may help to explain contradictory findings in research that showed no or reverse associations [31–34], by examining each professional burnout dimension separately. Our findings side with hypotheses in literature that those in COVID-19-related care experience a greater sense of accomplishment but experience a higher risk of burnout in other dimensions (i.c. depersonalisation in our study). One study also found higher levels of personal accomplishment among frontline COVID-19 workers, but found no differences on other burnout dimensions [32]. Despite academic burnout rates appearing more moderate than those of professional burnout, COVID-19-related experiences did show a relation with academic burnout. All academic burnout dimensions (higher EE and DP/CY and lower PA) in our study were significantly more pronounced when perceived impact of COVID-19 on their studies was higher. Some contradictory findings exist in literature, with some studies finding higher [35, 36, 45] and other no difference [37] in academic burnout after the pandemic. These higher burnout rates were related to students’ fears of missing essential learning opportunities [35, 36, 45] and missing social support [37]. It seems that how students’ individual experiences of the pandemic may cause variability in academic burnout risk.

### Qualitative findings on burnout experiences during COVID-19

The qualitative data indicated students and residents have a need: 1) to achieve necessary (learning) outcomes; 2) for clarity and knowing how to cope; and 3) for recognition and relatedness. These categories are in line with the necessary job resources of the JD-R model [8]. Each of these experiences and needs will be discussed next.

#### The need to achieve necessary (learning outcomes)

##### Demands

Students mentioned an increased workload, stress related to having to combine everything and a lack of adaptation of requirements to the new situation. It is clear that, certainly at the beginning of the pandemic, a mismatch has arisen between ‘regular/essential’ learning goals and the learning opportunities that were offered [45, 46]. Although new and important learning goals emerged related to pandemics and infectious care, the originally set goals cannot simply be overridden.

##### Resources

Academic training should nevertheless be able to adapt by using other ways to achieve learning goals, e.g., using virtual versions of history taking, examinations, decision-making, clinical case-or vignette-based learning and debate styles [47–49]. It has moreover been suggested to include pandemic preparation in the standard curriculum, incl. resilience building [50].

##### Strain

As in other studies [44, 45, 51–53], medical students or residents mentioned high emotional burden and psychological distress (e.g., stress, uncertainty, loneliness, exhaustion, powerlessness, and lack of motivation to study). The risk of mental health burden, especially when volunteering and helping out in COVID-19 related care, has been linked to a lack of pandemic preparation in their curriculum [50]. Participants also experienced stress related to seeing fewer learning opportunities, consistent with other studies [44, 48, 54, 55]. Many volunteered to help overcome the pandemic, partly also to compensate for this lack of learning opportunity. Students moreover feared they will later need to catch up on lost learning opportunities [48].

#### The need for clarity and knowing how to cope

##### Resources

Learning techniques to cope with challenges during their training can help the lifelong learning of skills to handle difficult career situations in later professional life [48]. A higher sense of coherence, characterized by considering challenges as comprehensible, manageable and meaningful, has shown to protect against academic burnout [56]. Such sense of coherence may be strengthened by perceptual interventions (e.g. mindfulness training) [57] and by behavioral interventions, such as empowerment in identifying resources to handle stressors [58].

#### The need for recognition and active involvement

##### Demands

Since first lockdown, online teaching tools are more established [59], teleconsulting is implemented and vaccination is widely available in Belgium. However, a major pitfall during a crisis is not paying enough attention to the training aspect. Students have indicated they want to take an active role in necessary adaptations to their medical training during COVID-19 [53]. They have moreover reported that collective student action during the COVID-19 crisis, for example by setting up a team of students that coordinates volunteering, can create a sense of empowerment, purpose, and connection [60].

##### Resources

Mentoring groups help students to proactively detect solutions, handle unpredictable situations, and find peer support and recognition. Such mentoring programs have shown to reduce burnout rates among medical students outside of the COVID-19 period [61]. Lastly, not only peer support and recognition are needed, participants also highlighted a need for more understanding and recognition from supervisors and educators for their efforts in this crisis. Supervisors and educators are recommended to provide frequent and clear communication, emotional support and explore meaningful ways with their students to contribute to solving the crisis [62], to enhance their wellbeing.

## Conclusion

This study aimed to examine academic and professional burnout among master (‘students’, ‘interns’) and advanced master students (‘residents’) in medicine and examine associations with perceived impact of the COVID-19 crisis and involvement in COVID-19 related care, using the Job Demands-Resources model. Findings showed moderate levels of professional burnout and low levels of academic burnout. Students involved in COVID-19-related care experienced a higher impact of COVID-19 on their studies as well as on their internships/residency; a higher level of depersonalisation or cynicism in their internships or residency; but no differences with those not in COVID-19 related care in relation to academic burnout. Those who perceived a higher impact of COVID-19 had higher burnout rates on all dimensions for their studies, but not for their internships or residency. Master and advanced master students mentioned experienced changes in demands, resources, and strain in relation to a need to achieve necessary (learning) outcomes; to the need for clarity and knowing how to cope; and the need for recognition and relatedness. Explained variance was low, suggesting other factors than those included in the study play an important role in academic and professional burnout.

### Strengths and limitations

This study had several limitations. First, a convenience sample was used which may not be representative for all medical students and residents in Belgium. A healthy worker effect may have occurred when using Facebook groups where curriculum info is shared and that may be more often visited by those who are coping with their studies and training. However, such groups are also used for social support and may therefore also be visited by those who struggled more with academic burnout [63]. We therefore consider a healthy worker effect as less likely here. The response rate was moreover low, which we assume to be related to the sampling method. The invitation may have easily been missed by students who did not frequently visit social media, or if many other posts occurred on the social media page. Raising awareness for the invitation by visiting students in their auditoria, may increase response rate but was not possible during lockdown. Moreover, Belgium is a country that is high on wealth and low on socioeconomic inequality, it is possible our results would not transfer to resource-strained countries. Second, the study examined the role of participating in COVID-19-related care, but no further detail was available on tasks performed in this care. Further research may wish to examine whether specific tasks (e.g., triage, administrative) are associated differently with burnout dimensions. Third, the study only focused on negative aspects (burnout) and not on positive aspects such as dedication, engagement, and vigor. Future research may wish to add the use of items that can tap into such positive dimensions of a work and learning experience. The explained variance of our included variables in predicting burnout was low, and these positive dimensions of a work and learning experience may help to further explain differences in burnout. Finally, it is important to note that this study was conducted during the first wave of the pandemic, which was an unexpected, unpredictable, and stressful situation for all parties, both on the ‘teaching’ and ‘learning’ side. Certain adaptations have been made since then, which might show different burnout rates and experiences. The study also has several strengths. Many studies on burnout among medical students do not distinguish between study-related burnout and work-related burnout. Our study used different scales to measure these forms of burnout, highlighting different results for academic and professional burnout. We used a combination of validated scales and open-ended questions, which led to a richness of data and the possibility to elucidate quantitative results. The focus on burnout dimensions rather than overall rates helped to better understand student and resident experiences. Finally, the study was carried out by a multidisciplinary team and led by a master student, which aided in designing more comprehensive conclusions and recommendations.

## Supplementary Information

**Additional file 1: Suppl. Table 1.** Descriptive statistics and correlations of the variables in the study (*n*=194). **Suppl. Table 2.** Comparison of burnout by calculation methods.

## Data Availability

The quantitative datasets used and/or analysed during the current study are available from the corresponding author on reasonable request. For reasons of confidentiality and as agreed in the ethical approval from our committee, the qualitative verbatim dataset is not available.
