# Supplementary material for: Experiences of academic and professional burn-out in medical students and residents during first COVID-19 lockdown in Belgium: a mixed-method survey
Source: BMC Med Educ. 2022 Aug 20;22:631. doi: 10.1186/s12909-022-03694-z (PMC9391213; doi:10.1186/s12909-022-03694-z)
Supplement: Supplementary file 1 — Additional file 1: Suppl. Table 1. Descriptive statistics and correlations of the variables in the study (n=194). Suppl. Table 2. Comparison of burnout by calculation methods. [file 12909_2022_3694_MOESM1_ESM.docx]

**Supplementary Material –**

The validation of MBI-SS by Faye-Dumanget et al. showed an internal consistency of *α* =.77 for the emotional exhaustion dimension (EE, 5 items, e.g. ‘I feel emotionally drained from my studies’); *α* = .72 for academic efficacy (AE, further referred to as PA, personal accomplishment for ease of comparison with professional burnout, 6 items, e.g. ‘In my opinion, I’m a good student’) and *α* = .81 for the cynicism or depersonalisation dimension (CY, 4 items, e.g. ‘I doubt the significance of my studies’) [1].

The validation by Dion & Tessier of the MBI-HSS showed an internal consistency of *α* = .90 for EE, *α* = .64 for DP, *α* = .74 for the PA. Internal consistency of the dimensions in our dataset were *α* = .89 for EE; *α* = .76 for DP and *α* = .77 for PA [2]. All dimensions showed good internal consistency.

**Suppl. Table 1**. Descriptive statistics and correlations of the variables in the study (n=194)

|  | **M** | **SD** | **1** | **2** | **3** | **4** | **5** | **6** | **7** | **8** |
| --- | --- | --- | --- | --- | --- | --- | --- | --- | --- | --- |
| **1. Age** | 24.95 | 2.53 |  |  |  |  |  |  |  |  |
| **2. MBI SS - Emotional exhaustion** | 19.15 | 4.62 | .083 |  |  |  |  |  |  |  |
| **3. MBI SS - Cynicism** | 11.48 | 4.54 | .151* | .651*** |  |  |  |  |  |  |
| **4. MBI SS - Personal accomplishment** | 22.15 | 4.15 | -.210** | -.463*** | -.516*** |  |  |  |  |  |
| **5. MBI HSS - Emotional exhaustion** | 25.96 | 11.35 | .111 | .662*** | .622*** | -.310** |  |  |  |  |
| **6. MBI HSS - Depersonalisation** | 9.87 | 6.53 | .148* | .304*** | .428*** | -.305** | .475** |  |  |  |
| **7. MBI HSS - Personal accomplishment** | 34.99 | 7.39 | -.108 | -.272*** | -.342** | .351** | -.300** | -.260** |  |  |
| **8. Perceived impact of COVID-19 on studies** | 4.35 | 1.52 | -.039 | .240** | -.195** | -.145* | .085 | .007 | -.135 |  |
| **9. Perceived impact of COVID-19 on internship** | 4.03 | 1.70 | .039 | .033 | .068 | -.085 | .028 | .042 | .010 | .589*** |

* *p*<0.05; ** *p*<.01; *** *p*<.001

**Suppl. Table 2.** Comparison of burnout by calculation methods

|  | **Professional burnout** | | **Academic burnout** | |
| --- | --- | --- | --- | --- |
|  | **Current study** | **Previous studies** | **Current study** | **Previous studies** |
| **Based on continuous scores on subdimensions** | | | | |
| **EE** | M=25.96 ± 11.35 | M=22.93 ± 10.25*** [3] | M=19.15 ± 4.62 | M=27.50 ± 7.16 *** [4] |
| **PA** | M=34.99 ± 7.39 | M=35.11 ± 8.03 [3] | M=22.12 ± 4.15 | M=22.38 ± 6.89 [4] |
| **CY / DP** | M=9.87 ± 6.53 | M=8.88 ± 5.64* [3] | M=11.48 ± 4.54 | M=14.83 ± 7.09 *** [4] |
| **Based on overall burnout rated calculated with cut-off points** | | | | |
| **Two-dimensional (high on EE OR on CY/DP)** | 61.9% | 52.7% [5] | 24.7% | 22.6% [6]  44.9% [7] |
| **Two-dimensional (high on EE AND high on CY/DP)** | 28.9% | 26.7% [8] | 9.3% | No comparison found |
| **Three-dimensional (high on EE, AND high on CY/DP, AND low on PA)** | 17.0% | 12.5% [9]  15.1% [10]  7.53% [11], as reported in [3] | 0.5% | 26.4% [7] |

Note: Comparisons with other studies on overall burn-out rates are listed with some studies as examples. Unfortunately, systematic review findings either reported no summary rate but a range [3], or created a summary rate across several tools and calculation methods [12, 13].

Significance of t-tests comparing our means with those found in other studies: *** p<.001, ** p<.01, * p<.05

Bibliography

1. Faye‐Dumanget C, Carré J, Le Borgne M, Boudoukha PAH: **French validation of the Maslach Burnout Inventory‐Student Survey (MBI‐SS)**. *Journal of evaluation in clinical practice* 2017, **23**(6):1247-1251.

2. Dion G, Tessier R: **Validation and translation of the burnout inventory of Maslach and Jackson**. *Canadian Journal of Behavior Science* 1994, **26**:210-227.

3. Erschens R, Keifenheim KE, Herrmann-Werner A, Loda T, Schwille-Kiuntke J, Bugaj TJ, Nikendei C, Huhn D, Zipfel S, Junne F: **Professional burnout among medical students: systematic literature review and meta-analysis**. *Medical teacher* 2019, **41**(2):172-183.

4. Gil-Calderón J, Alonso-Molero J, Dierssen-Sotos T, Gómez-Acebo I, Llorca J: **Burnout syndrome in Spanish medical students**. *BMC medical education* 2021, **21**(1):1-7.

5. Dyrbye LN, Eacker A, Durning SJ, Brazeau C, Moutier C, Massie FS, Satele D, Sloan JA, Shanafelt TD: **The impact of stigma and personal experiences on the help-seeking behaviors of medical students with burnout**. *Academic medicine* 2015, **90**(7):961-969.

6. Galán F, Sanmartín A, Polo J, Giner L: **Burnout risk in medical students in Spain using the Maslach Burnout Inventory-Student Survey**. *International archives of occupational and environmental health* 2011, **84**(4):453-459.

7. Boni RAdS, Paiva CE, De Oliveira MA, Lucchetti G, Fregnani JHTG, Paiva BSR: **Burnout among medical students during the first years of undergraduate school: Prevalence and associated factors**. *PloS one* 2018, **13**(3):e0191746.

8. Cecil J, McHale C, Hart J, Laidlaw A: **Behaviour and burnout in medical students**. *Medical education online* 2014, **19**(1):25209.

9. Legassie J, Zibrowski EM, Goldszmidt MA: **Measuring resident well-being: impostorism and burnout syndrome in residency**. *Journal of general internal medicine* 2008, **23**(7):1090-1094.

10. Popa-Velea O, Diaconescu L, Mihăilescu A, Jidveian Popescu M, Macarie G: **Burnout and its relationships with alexithymia, stress, and social support among Romanian medical students: a cross-sectional study**. *International journal of environmental research and public health* 2017, **14**(6):560.

11. Bugaj TJ, Müksch C, Ehrenthal JC, Köhl-Hackert N, Schauenburg H, Huber J, Schmid C, Erschens R, Junne F, Herzog W: **Stress in medical students: a cross-sectional study on the relevance of attachment style and structural integration**. *Psychotherapie, Psychosomatik, Medizinische Psychologie* 2016, **66**(2):88-92.

12. Low ZX, Yeo KA, Sharma VK, Leung GK, McIntyre RS, Guerrero A, Lu B, Sin Fai Lam CC, Tran BX, Nguyen LH: **Prevalence of burnout in medical and surgical residents: a meta-analysis**. *International journal of environmental research and public health* 2019, **16**(9):1479.

13. Frajerman A, Morvan Y, Krebs M-O, Gorwood P, Chaumette B: **Burnout in medical students before residency: a systematic review and meta-analysis**. *European Psychiatry* 2019, **55**:36-42.
